# Vernal pool amphibian breeding ecology monitoring from 1931 to present: A harmonised historical and ongoing observational ecology dataset

**DOI:** 10.3897/BDJ.8.e50121

**Published:** 2020-04-14

**Authors:** Alexis Garretson, Megan Napoli, Natalie Feldsine, Penelope Adler-Colvin, Elizabeth Long

**Affiliations:** 1 George Mason University, School of Systems Biology, Fairfax, United States of America George Mason University, School of Systems Biology Fairfax United States of America; 2 George Mason University, Department of Biology, Fairfax, United States of America George Mason University, Department of Biology Fairfax United States of America; 3 Mohonk Preserve, New Paltz, United States of America Mohonk Preserve New Paltz United States of America; 4 SUNY New Paltz, New Paltz, United States of America SUNY New Paltz New Paltz United States of America

**Keywords:** amphibians, frogs, salamanders, phenology, wood frog, spring peeper, spotted salamander, Jefferson salamander, red-spotted newt, seasonal wetlands, vernal pools, ecology, community ecology, Mohonk Preserve, Daniel Smiley Research Center, Shawangunk Ridge, water quality, temperature, pH, dissolved oxygen, conductivity, nitrate, reproductive ecology, breeding, seasonality

## Abstract

**Background:**

For 88 years (1931-present), the Mohonk Preserve's Daniel Smiley Research Center has been collecting data on occupancy and reproductive success of amphibian species, as well as associated water quality of 11 vernal pools each spring (February to May). Though sampling effort has varied over the dataset range, the size of the dataset is unprecedented within the field of amphibian ecology. With more than 2,480 individual species sampling dates and more than 151,701 recorded individual occurrences of the nine amphibian species, the described dataset represents the longest and largest time-series of herpetological sampling with paired water quality data.

**New information:**

We describe the novel publication of a paired dataset of amphibian occurrence with environmental indicators spanning nearly 90 years of data collection. As of February 2020, the dataset includes 2,480 sampling dates across eleven vernal pools and 151,701 unique occurrences of egg masses or individuals recorded across nine species of amphibian. The dataset also includes environmental conditions associated with the species occurrences with complete coverage for air temperature and precipitation records and partial coverage for a variety of other weather and water quality measures. Data collection has included species, egg mass and tadpole counts; weather conditions including precipitation, sky and wind codes; water quality measurements including water temperature and pH; and vernal pool assessment including depth and surface vegetation coverage. Collection of data was sporadic from 1931–1991, but data have been collected consistently from 1991 to present. We also began monitoring dissolved oxygen, nitrate concentrations and conductivity of the vernal pools using a YSI Sonde Professional Plus Instrument and turbidity using a turbidity tube in February 2018. The dataset (and periodic updates), as well as metadata in the EML format, are available in the Environmental Data Initiative Repository under package edi.398.

## Introduction

**Figure 1.**
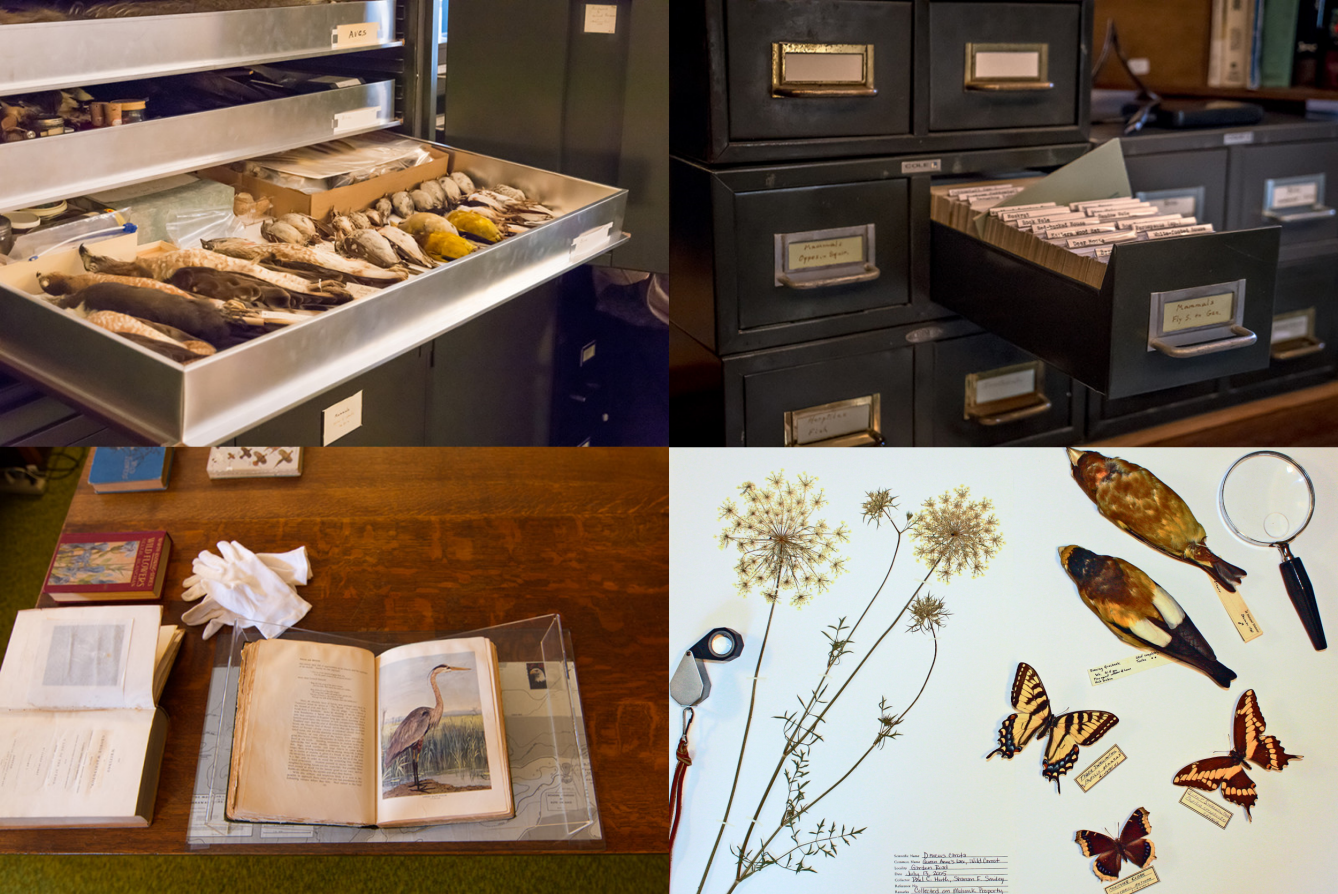
The Daniel Smiley Research Center (DSRC) collection of long-term research data includes more than 14,000 index cards with historical observations (top right), a significant library of historical reports and natural history literature (bottom left) and more than 60,000 physical items, including 107 bird specimens (top left, bottom right).

Mohonk Preserve is a nature preserve and land trust located in New Paltz, New York State, USA. As the largest non-profit nature preserve in New York State, Mohonk Preserve protects and manages more than 8,000 acres of the Shawangunk Mountains, a northern section of the Appalachian Mountains. Renowned for its high biodiversity value, the Shawangunks harbour more than 1,400 known plant and animal species, including 57 that are rare and imperilled (Thompson and Huth 2011, Batcher 2000). Mohonk Preserve's conservation science division is affiliated with the Organization of Biological Field Stations and acts as a NOAA Climate Observation Center. Mohonk Preserve's conservation unit is the Daniel Smiley Research Center (DSRC), which coordinates a research and educational network that includes over 20 researchers from regional and national research institutions and more than 1,000 students and dozens of faculties from colleges and universities in the Hudson Valley and beyond. DSRC staff and citizen scientists carry out a variety of long-term monitoring projects, including the described amphibian breeding in woodland vernal pools. In addition to the ongoing monitoring, the DSRC manages an extensive archive of historical observations including 86 years of natural history observations, 123 years of daily weather data, 60,000 physical items, 9,000 photographs and a research library. Of those physical items, there are over 3,000 herbarium specimens, 139 mammal specimens, 107 bird specimens, 140 butterfly specimens, 400 arthropod specimens and over 14,000 index cards with handwritten and/or typed natural history observations. The data and natural history collections underpin the Mohonk Preserve’s land management and stewardship (Fig. 1) and have been crucial to an increasing number of scientific publications (e.g. Cook et al. 2009, Cook et al. 2008, Charifson et al. 2015, Richardson et al. 2016).

The Vernal Pool Monitoring programme began in 1931 with the observations of Daniel Smiley (1907–1989). Smiley documented extensive records of a variety of taxa within the Shawangunk Mountains beginning in the late 1920s (Huth 1996). The majority of his observations are recorded in a card filing system or one- to two-page reports, that are now available in the DSRC archives. He first began monitoring amphibians in 1930 and began regularly monitoring vernal pools in the surrounding regions in the 1950s. Smiley's particular interest in the impacts of acid rain and pesticides provides us with records of water quality extending to some of Smiley's earliest observations (Huth 1996, Weathers et al. 1986).

**Figure 2.**
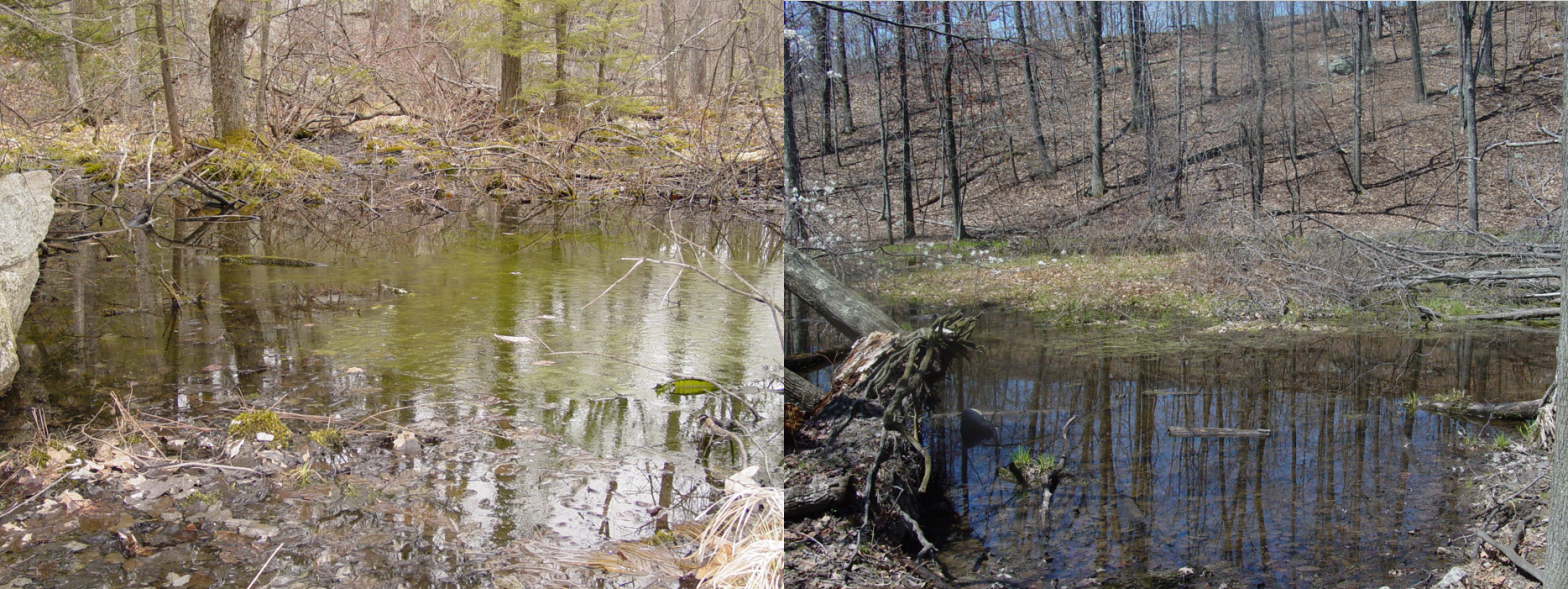
Representative images of two of the sampled vernal pools: Sleepy Hollow (left) and Canaan (right).

The presented dataset includes observations at 11 vernal pools on Mohonk Preserve lands: Sleepy Hollow, Ski Loop, Canaan, Long Woodland Swamp, Long Woodland Pool, Talus, Terrace, Bonticou, Oakwood, Hermits and North Mud Pond (Fig. 2). These vernal pools vary in size and are distributed across the Mohonk Preserve landscape at a range of elevations (166–384 m). The current goal of the project is to monitor the seasonality and reproductive ecology of amphibians. As the breeding behaviour of amphibians is highly dependent on environmental quality, particularly water quality, data collection in recent years has strived to provide a holistic environmental context for occurrence records (Semlitsch 2002, Marco et al. 1999, Karraker et al. 2008, Hamer and McDonnell 2008).

**Figure 3.**
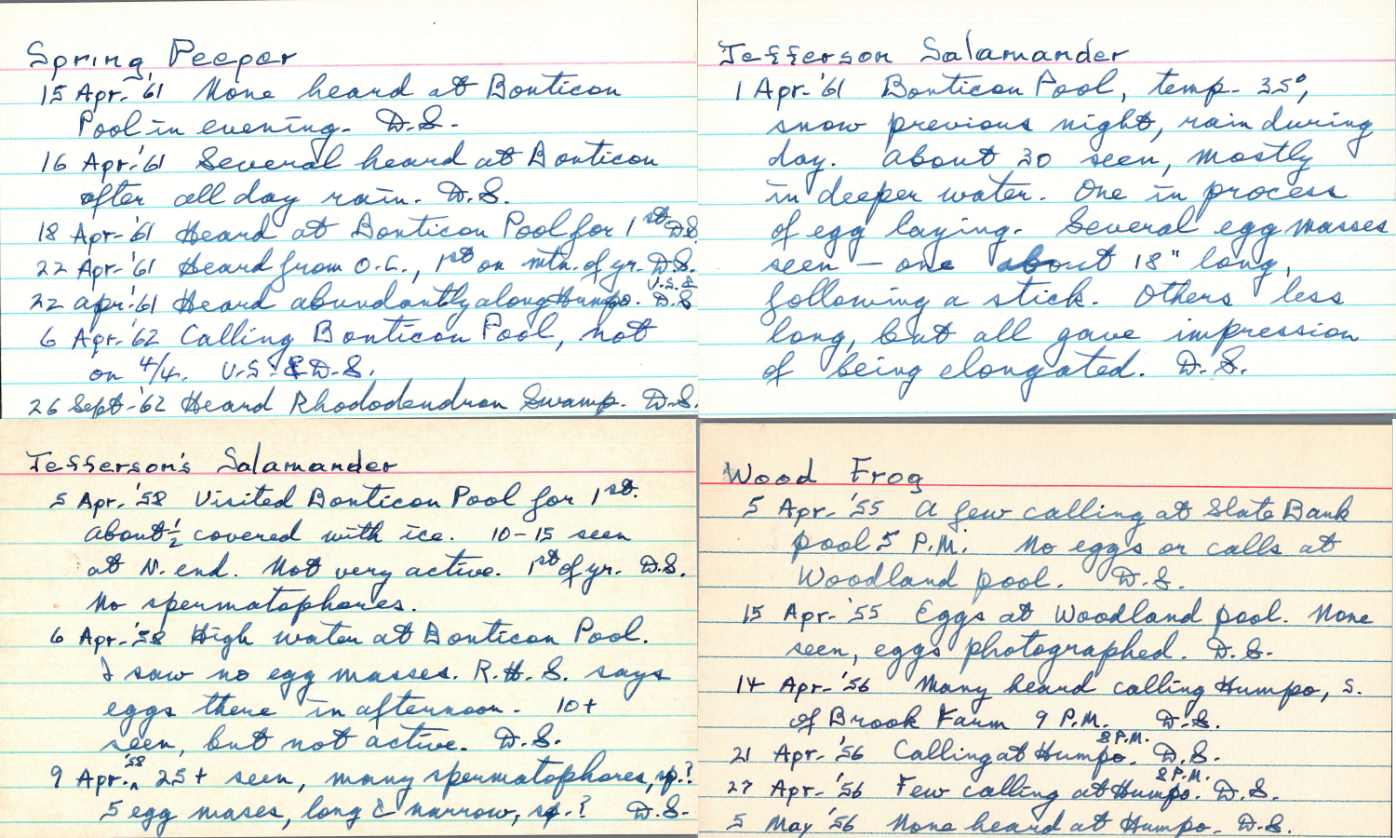
Scanned historical occurrence notecards. Dates, locations, occurrence stages and any water quality or environmental indicators were extracted from narrative statements and are included in the described dataset.

A major hurdle to making this natural history dataset available to the research community has been the digitisation of the historical data records. The majority of the historical data points were extracted from the notecards (Fig. 3) These cards were scanned, transcribed and formatted for data archiving by volunteer citizen scientists at the DSRC facilities, demonstrating the significant impact of citizen science contributions in natural history data rescue efforts and mitigating data risk factors (Griffin 2017, Brunet and Jones 2011, Mayernik et al. 2020). Due to the size and scope of the DSRC's historical holdings, the digitisation process remains ongoing and, as historical occurrences become available, they will be added to the described dataset periodically. In addition to the digitised historical data, the dataset is also comprised of ongoing, yearly vernal pool monitoring. Combining the historical data with ongoing monitoring enables researchers to elucidate long-term trends, project impacts of climate change or urbanisation and compare these predictions with future and current data collection.

This dataset significantly adds to our knowledge of amphibian breeding ecology by providing a nearly 90-year time series of amphibian reproductive occurrences paired with environmental variables from a single geographic region with multiple replicate pools. With more than 2,480 individual species sampling dates and more than 151,701 recorded individual occurrences of the nine amphibian species, the described dataset represents the longest and largest time-series of herpetological sampling with paired water quality data. Additionally, the dataset incorporates records from Long Woodland Vernal Swamp from years immediately preceding its drying. These records may be of particular interest to researchers interested in transitional ecology. The dataset also extends into the 1930s, allowing for significant investigations into the impacts of climate change and acid rain on amphibian reproductive ecology and phenology. In this paper, we will describe the data structure and synthesise the collection protocols in the hopes of facilitating future reuse and consistent access to this valuable data resource.

## Sampling methods

### Sampling description

Amphibian occupancy, breeding and water quality of vernal pools on the Preserve have been monitored since 1931. Sampling effort has varied significantly over the dataset range, with stochastic sampling prior to 2017 and post-2017 weekly spring sampling. From 1931 - 2015, data collected at each vernal pool varied, but often included current weather conditions, water level (%), water temperature (°F), water pH and species/egg mass counts. Observations at each vernal pool occur annually between February and May. From 1931 – 1991, sampling at vernal pools and the number of vernal pools sampled each year varied. Starting in 1991, each vernal pool was observed at least two times each spring. Starting in 2016, a more rigorous protocol was adopted from the USGS Amphibian Reproductive Monitoring Initiative programme with sampling at 10 vernal pools being completed at least four times per spring season (Muths et al. 2005).The number of observers during each survey varies and is weather and logistic dependent, but with a goal of weekly sampling of each vernal pool, beginning at the onset of amphibian emergence and continuing until larvae hatch from egg masses.

In 2016, the new protocol dictated more parameters to be sampled consistently. Upon arrival at each vernal pool, initial observations performed included:

1. weather conditions: sky code, wind code, air temperature (°F) and previous day precipitation occurrence; and
2. qualitative measurements: water level (%), surface ice (%), surface vegetation (%), surface vegetation species, water odour and fairy shrimp (order Anostraca) presence.

In addition to the qualitative observations, quantitative measurements are taken of:

1. water quality: temperature (°F), pH, conductivity (µS), dissolved oxygen (%), nitrate concentration (mg/l), and turbidity (NTU); and
2. amphibian species counts: number of adults alive, dead, in amplexus; egg masses, calling frogs, spermatophores, juveniles and tadpole/larvae.

**Figure 4.**
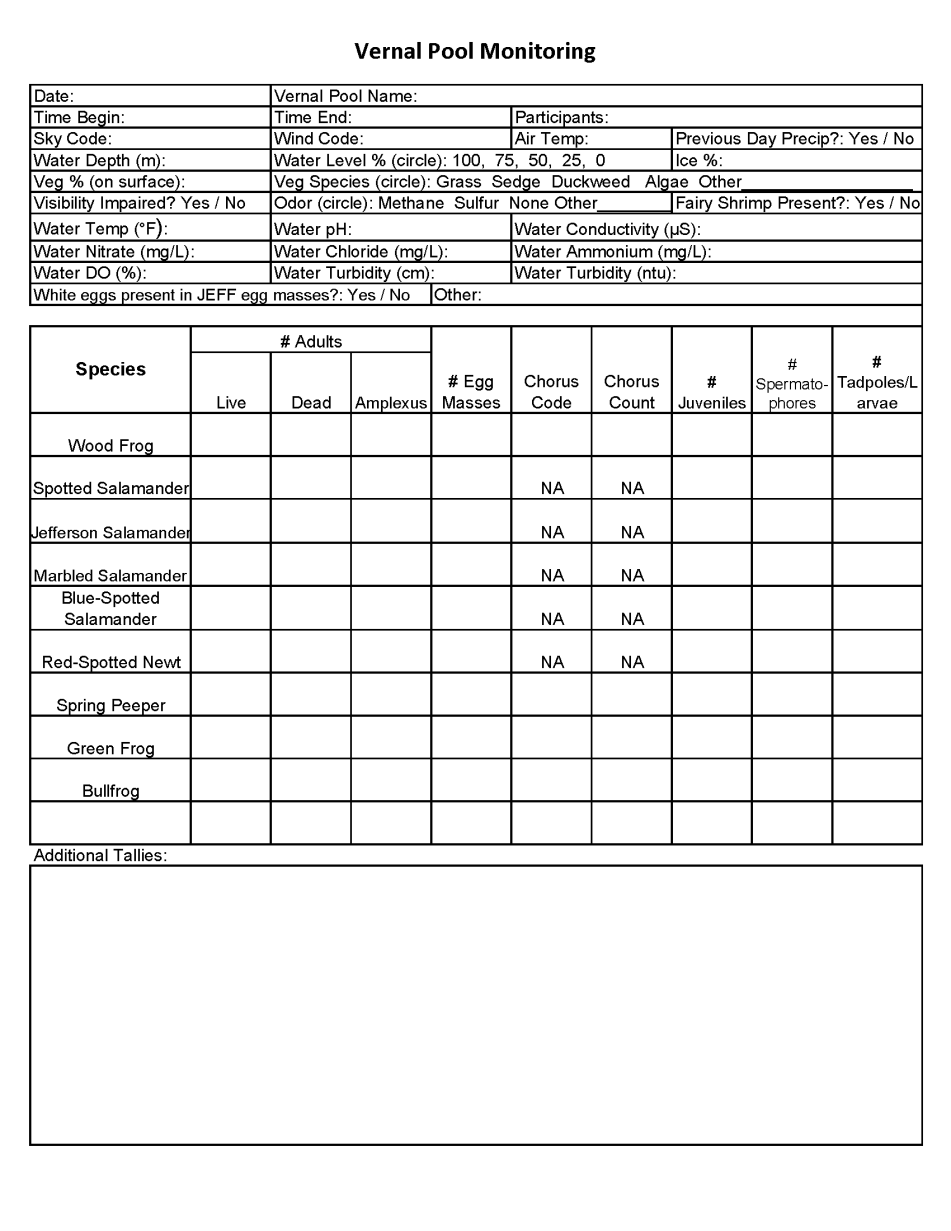
Current format of the vernal pool monitoring data sheet for ongoing data collection. As additional data are collected, the data object in the Environmental Data Initiative repository (package identifier: edi.398) will be updated accordingly.

The data are recorded by hand on to standardised data sheets (Fig. 4) and later input into a machine-readable format. All parameters of amphibian species counts since 2016 were collected using the Double-Observer Dependent method (Cook and Jacobson 1979, Nichols et al. 2000). Observer A walks halfway around the pool and calls out counts to Observer B. Observer B records count made by Observer A, but also records counts that Observer A missed without informing Observer A. Observers A and B switch roles when the second half of the pool is surveyed. Prior to November 2017, water from the vernal pool was collected in a high-density polyethylene bottle and temperature was immediately recorded on-site with an analogue thermometer. The sample was then brought back to the DSRC where pH was measured. Prior to 13 December 1991, pH was measured using a Sargent-Welch analogue pH meter. Beginning 13 December 1991, pH was measured with a Fisher Accumet digital pH meter with higher resolution and probes were replaced every 6 months. In 2002, the meter was replaced with a new Fisher Accumet pH meter. Starting in February 2018, temperature and pH were collected in situ using a YSI Sonde Professional Plus hand-held multiparameter meter and additional Sonde measurements of percent dissolved oxygen, nitrate concentrations and conductivity were incorporated in the water quality measurements. Starting in March 2019, a turbidity tube was incorporated to measure turbidity.

### Quality control

Prior to the publication of the dataset, historical records were checked for biological consistency. During field surveys, utmost caution was used when monitoring and sampling from the vernal pools. To avoid disturbance, wading was not done in the pools and the YSI Sonde was only submerged in areas that did not contain visible animals, egg masses, spermatophores or larvae. Starting in 2017, between each vernal pool survey, equipment and boots were disinfected with a 5% bleach solution to avoid transference of disease. The YSI Sonde was calibrated before each vernal pool survey to ensure that water quality measurements were accurate; probes were replaced as needed.

## Geographic coverage

### Description

**Figure 5.**
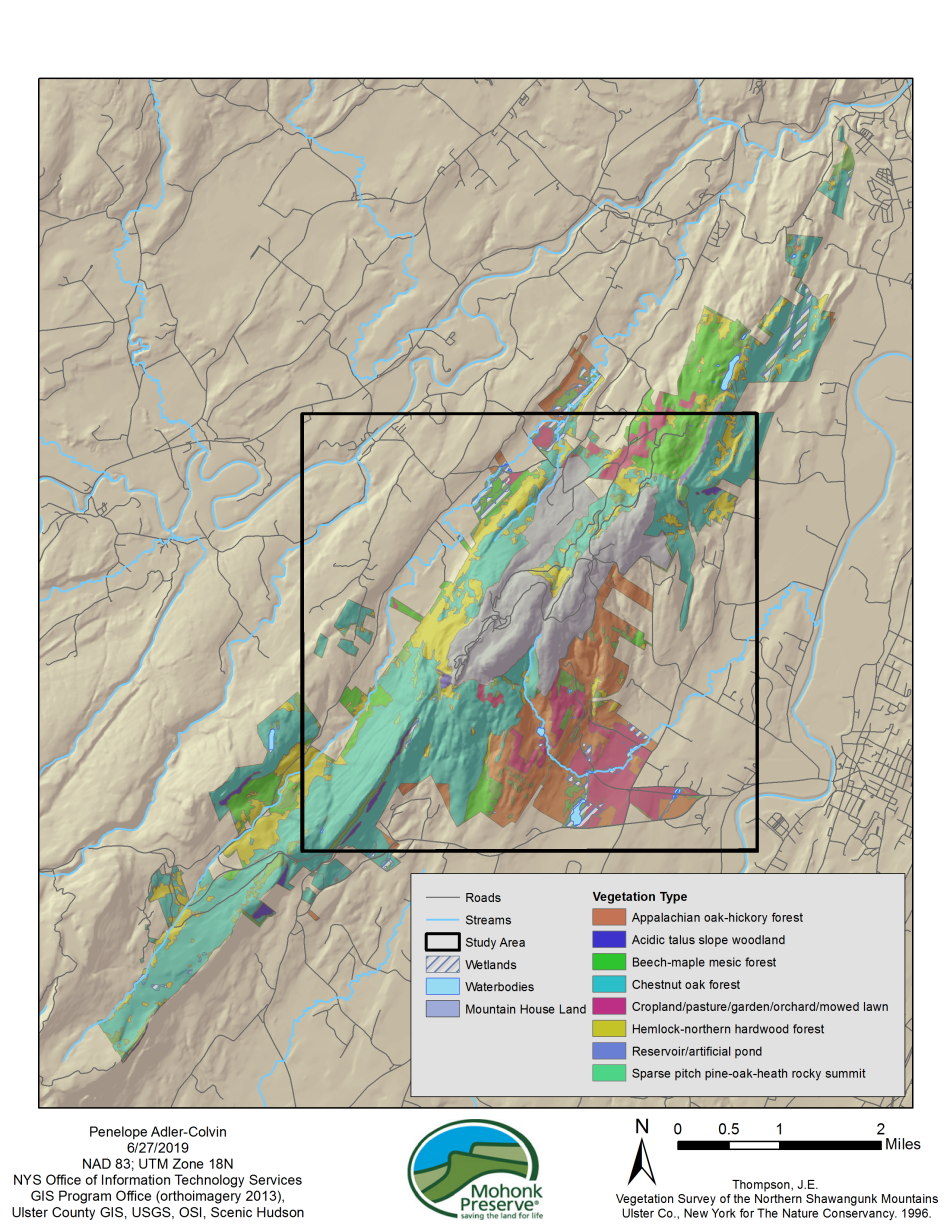
Vegetation cover and geography of the vernal pool data collection region in the Mohonk Preserve. Vegetation cover adapted from Thompson and Huth (2011) and New York State Museum (1999).

Mohonk Preserve is located in the Hudson Valley region of New York State, in Ulster County. The Preserve has land in the towns of New Paltz, Gardiner, Rochester, Marbletown and Rosendale. Main vegetation types of the Preserve are Appalachian oak-hickory forest, beech-maple mesic forest, chestnut oak forest, hemlock northern hardwood forest and grasslands/pastures (Fig. 5, Thompson and Huth 2011). The geology of this area has been influenced by the Taconic and Acadian orogenies; rocks occurring in abundance include shale, greywacke, quartz conglomerate and sandstone. Surficial geology of the Preserve includes kame deposits, lacustrine deltas, lacustrine silt & clay, bedrock and till. Bedrock geology includes Normanskill Shale and the Bloomsburg Formation (New York State Museum 1999). The coordinates below designate the bounding box surrounding the area where observations were recorded. Due to the sensitivity of the vernal pools and the amphibian species, specific locations were not revealed as part of this data package, but are available upon request from the DSRC to individuals carrying out direct research.

### Coordinates

41.80133 and 41.738856 Latitude; -74.109075 and -74.190815 Longitude.

## Taxonomic coverage

### Description

**Figure 6.**
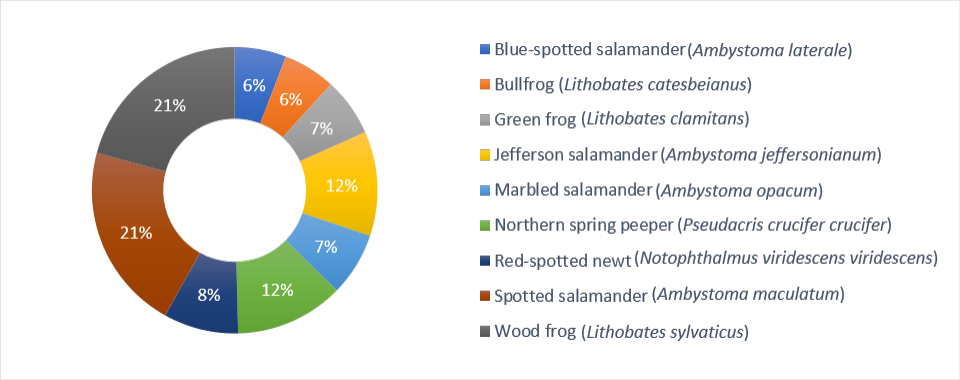
Pie chart showing the percent of dataset occurrence records by species. Jefferson/blue-spotted salamander complex omitted from the total, with 13 total records (0.5%).

The described dataset, ongoing as of February 2020, includes 2,480 recorded dates, sampled across the nine amphibian species. Spotted salamanders were surveyed on the greatest number of dates, with 521 unique events and blue-spotted salamanders were surveyed the least, with 144 unique events (Fig. 6). Across all species, life stages and sampling events, a total of 151,701 individuals were recorded. All the species included in the sampling are native to this region. Three of the sampled *Ambystoma* species: Jefferson salamander, marbled salamander and blue-spotted salamander were listed in 2013 as species of special concern in the State of New York as defined in Section 182.2(i) of 6NYCRR Part 182. As defined by the State, species of special concern need increased consideration and monitoring, but current information does not warrant listing these species as threatened or endangered.

The current taxonomic authority of the dataset is the Integrated Taxonomic Information System (ITIS) (Bisby et al. 2006). If the ITIS taxonomic classification of the monitored species changes, the dataset will be updated at that point to reflect those changes. Particularly, there has been significant debate about the appropriate genus name for green frogs, wood frogs and bullfrogs. The elevation of *Lithobates* as a genus name for these species has been questioned by Yuan et al. (2016) and Pauly et al. (2009). Presently, *Rana* is used by the vast majority of ranid systematists around the world, so we anticipate the eventual transition from *Lithobates* to *Rana* in ITIS in the near future and the subsequent updating of the data package.

### Taxa included

**Table:** 

Rank	Scientific Name	Common Name
species	*Ambystoma maculatum* (Shaw, 1802)	Spotted Salamander
species	*Ambystoma jeffersonianum* (Green, 1827)	Jefferson Salamander
species	*Ambystoma laterale* (Hallowell, 1856)	Blue-spotted Salamander
species	*Notophthalmus viridescens viridescens* (Rafinesque, 1820)	Red-spotted Newt
species	*Pseudacris crucifer crucifer* (Wied-Neuwied, 1838)	Northern Spring Peeper
species	*Lithobates clamitans* (Latreille in Sonnini de Manoncourt and Latreille, 1801)	Green Frog
species	*Lithobates catesbeianus*	Bullfrog
species	*Ambystoma opacum* (Gravenhorst, 1807)	Marbled Salamander
species	*Lithobates sylvaticus* (LeConte, 1825)	Wood Frog

## Temporal coverage

### Notes

The temporal extent of the dataset is from 1931-04-21 through 2019-05-01; and the months included in the dataset are February - May. Data collection is ongoing, as is digitisation of old records. Thus, the dataset will be updated accordingly over time and will continue to be available under edi.398 in the Environmental Data Initiative repository (Preserve et al. 2019, Fig. 4). Collection of both species occurrence data and environmental data was sporadic from 1931 - 1991, but spring data collection from 10 pools has been consistent from 1991 - present (Fig. 7). If pools dry up or access is no longer possible, additional pools may be added to have coverage of 10 pools each spring. For example, Long Woodland Vernal Swamp was no longer holding water as of 2017, so consistent collection of data at Ski Loop Vernal Pool commenced in 2018.

## Usage rights

### Use license

Creative Commons Public Domain Waiver (CC-Zero)

### IP rights notes

This data package is released to the “public domain” under Creative Commons CC0 1.0 “No Rights Reserved” (see: https://creativecommons.org/publicdomain/zero/1.0/). It is considered professional etiquette to provide attribution of the original work, if this data package is shared in whole or by individual components. A generic citation is provided for this data package on the website https://portal.edirepository.org (herein “website”) in the summary metadata page. Communication (and collaboration) with the creators of this data package is recommended to prevent duplicate research or publication. This data package (and its components) is made available “as is” and with no warranty of accuracy or fitness for use. The creators of this data package and the website shall not be liable for any damages resulting from misinterpretation or misuse of the data package or its components. Periodic updates of this data package may be available from the website.

## Data resources

### Data package title

Mohonk Preserve Amphibian and Water Quality Monitoring Dataset at 11 Vernal Pools from 1931–Present

### Resource link

https://doi.org/10.6073/pasta/9eb31b2e15146e6a71f3fea8d3724aaa 

### Alternative identifiers

Mohonk Vernal Pool Monitoring, edi.398

### Number of data sets

4

### Data set 1.

#### Data set name

Species occurrence data

#### Data format

Comma-separated values

#### Number of columns

16

#### Character set

UTF-8

#### Download URL

https://portal.edirepository.org/nis/dataviewer?packageid=edi.398.4&entityid=737389d6c2090ee27a3084898f2e7853

#### Description

This dataset includes the recorded observations of nine species of amphibians at 11 vernal pools. On some dates, no individuals were observed, but were looked for, so the column may include all 0 values. The individuals observed were noted by life stage, as described in the columns below. Finally, the chorus heard during sampling was recorded with a chorus code and a count of the number of calling individuals, if the calls were distinguishable.

**Data set 1.** 

Column label	Column description
CommonName	Common name of the amphibian species observed
ScientificName	Scientific name of the vernal pool amphibian species observed
Authorship	Citation for the scientific name
ITIS_TSN	The taxonomic serial number for the amphibian species in the Integrated Taxonomic Information System
NY_Concern	A binary variable indicating whether the species is presently listed as a New York state species of concern (1 = yes, 0 = no)
Location	Name of vernal pool for occurrence record
Sample_Date	Date of sample observation and occurrence record, format: YYYY-MM-DD
ChorusCode	Frog chorus code heard during sampling: 0 = No calling frogs; 1 = Individuals can be counted, calls not overlapping; 2 = Calls overlap (simultaneous calling), but individuals are distinguishable; 3 = Full chorus, calls continuous and overlapping. Cannot distinguish individuals; 4 = calling indicated, but additional information not provided (Historical code)
ChorusCount	If calls are distinguishable, the number of calling individuals.
Live_n	Number of live adults observed
Dead_n	Number of dead adults observed
AmplectantPairs_n	Number of amplectant pairs of adults observed
EggMass_n	Number of egg masses observed
Juv_n	Number of juvenile individuals observed
Sperm_n	Number of spermatophores observed
TadLarv_n	Number of tadpoles and larvae observed

### Data set 2.

#### Data set name

Weather and water quality data

#### Data format

Comma-separated values

#### Number of columns

22

#### Character set

UTF-8

#### Download URL

https://portal.edirepository.org/nis/dataviewer?packageid=edi.398.4&entityid=5835a29e0fbe1ba179ee5ffe27121df6

#### Description

This dataset includes environmental and water quality data points collected for each pool for each sampling date. There is 100% coverage of air temperature and the presence of previous day precipitation for all sites on all sampling dates, but the collection of other environmental indicators was inconsistent from 1931–1991. Since 1991, collection of these data points has been more consistent, with a significant increase in the number of variables available in Feb 2018 due to the continued use of the YSI Sonde Professional Plus instrument to collect water quality data.

**Data set 2.** 

Column label	Column description
Location	Name of vernal pool sampled
Sample_Date	Date of sample observations
Sky_Code	Categorical descriptor of sky conditions: 0 = Clear or few clouds (< 20% of sky); 1 = Partly cloudy or variable (20-50% of sky); 2 = Cloudy or overcast (> 50% of sky); 3 = Fog; 4 = Mist; 5 = Showers or light rain; 6 = Heavy rain; 7 = Sleet/hail; 8 = Snow
Wind_Code	Categorical descriptor of wind conditions: 0 = Calm, smoke rises vertically (< 1 mph); 1 = Light air movement, smoke drifts (2–3 mph); 2 = Light breeze, wind felt on face, leaves rustle (4–7 mph); 3 = Gentle breeze, leaves twigs in constant motion, raises dust (8–12 mph); 4 = Moderate breeze, small branches move (13–18 mph); 5 = Fresh breeze, small trees begin to sway (19–24 mph); 6 = Strong breeze, large branches move (25–31 mph); 7 = Strong winds (> 31 mph)
PDP	Binary variable for the presence of precipitation on the previous day (0 = no previous day precipitation, 1 = previous day precipitation)
Air_Temp_C	Air temperature at time of observation (degrees Celsius)
Water_Temp_C	Water temperature of the vernal pool at time of collection (degrees Celsius)
pH	pH of vernal pool water
Water_Depth	Quantitative depth measurement of the depth of the vernal pool (cm)
Water_Level	Qualitative measurement of how full the vernal pool was at the time of observation :0 = Pool is dry; < 25 = < 1/4 full relative to the maximum depth; 25 = 1/4 full relative to the maximum depth; 50 = 1/2 full relative to the maximum depth; 75 = 3/4 full relative to the maximum depth; 100 = full at the maximum depth.
Visibility_Imp	Binary variable designating whether visibility was impaired while counting egg masses or determining species identity (1 = yes, 0 = no)
Surface_Ice	Percentage of the vernal pool surface covered in ice
Surface_Veg	Percentage of the vernal pool surface covered by vegetation
SurfaceVeg_spp	Character string of vegetation species covering the surface of the vernal pool
Odor	Categorical variable for the odour emitted by vernal pool: None = no water odour; Peat = Peat water odour; Sulphur = Sulphur water odour; Methane = Methane water odour
Shrimp	Binary variable for the presence or absence of fairy shrimp in the vernal pool (1 = presence, 0 = absence)
Snails	Binary variable for the presence or absence of freshwater snails in the vernal pool water (1 = presence, 0 = absence)
Turbidity_NTU	Turbidity of the vernal pool water (nephlometric turbidity units)
Chloride	Chloride of the vernal pool water (milli-equivalents per litre)
Conductivity	Conductivity of the vernal pool water (Siemens per metre)
DO	Dissolved oxygen of the vernal pool water (milligrams per litre)
Nitrate	Nitrate of the vernal pool water (milligrams per litre)

### Data set 3.

#### Data set name

Additional location information

#### Data format

Comma-separated values

#### Number of columns

5

#### Character set

UTF-8

#### Download URL

https://portal.edirepository.org/nis/dataviewer?packageid=edi.398.4&entityid=ceaabc36d68cdfb681122ce1f0941420

#### Description

This dataset includes additional information about the 11 sampling locations included in the dataset, including the name used in the weather and water dataset and the species occurrence dataset and associated information about the vernal pool location and sizes. These data were collected in 1999 and we anticipate an update within the next few sampling years (Barbour 1999).

**Data set 3.** 

Column label	Column description
Location	Vernal pool location name
Elevation	Elevation of the vernal pool (metres)
MaxDepth	The maximum depth of the vernal pool at full capacity (metres)
Length	The length of the vernal pool at its longest point (metres)
Width	The width of the vernal pool at its widest point (metres)

### Data set 4.

#### Data set name

Metadata for Mohonk Preserve Amphibian and Water Quality Monitoring Dataset at 11 Vernal Pools from 1931-Present

#### Data format

Ecological Metadata Language

#### Number of columns

1

#### Character set

XMLENCOD

#### Download URL

https://portal.edirepository.org/nis/metadataviewer?packageid=edi.398.4&contentType=application/xml

#### Data format version

EML version 2.1.1

#### Description

The metadata, in the Ecological Metadata Language format as XML, for the described datasets and sampling project.

**Data set 4.** 

Column label	Column description
NA	NA

## Additional information

### Additional Data Considerations and Information

1. Due to the sensitivity of the vernal pools and the amphibian species, specific locations were not revealed as part of this data package, but are available upon request from the DSRC to individuals carrying out direct research.
2. Additional historical data exists in Daniel Smiley Research Center Library and is currently being digitised and will be added to the dataset as records become available.
3. Egg masses belonging to the Jefferson salamander or Jefferson/Blue-spotted salamander hybrids are incredibly difficult to distinguish. Historical determinations have been preserved as they were recorded, but additional care should be taken with these records.
4. Historical water depth measurements were taken as inches from maximum pool depth, so prior to 2015, depth measurements were converted to cm and added to the maximum depths recorded in the location information. Any remaining negative depths were omitted for clarity.
5. Long Woodland Swamp data collection ceased in 2016 due to the swamp drying up and the pool no longer holding water in the spring.

## Figures and Tables

**Figure 7a.**
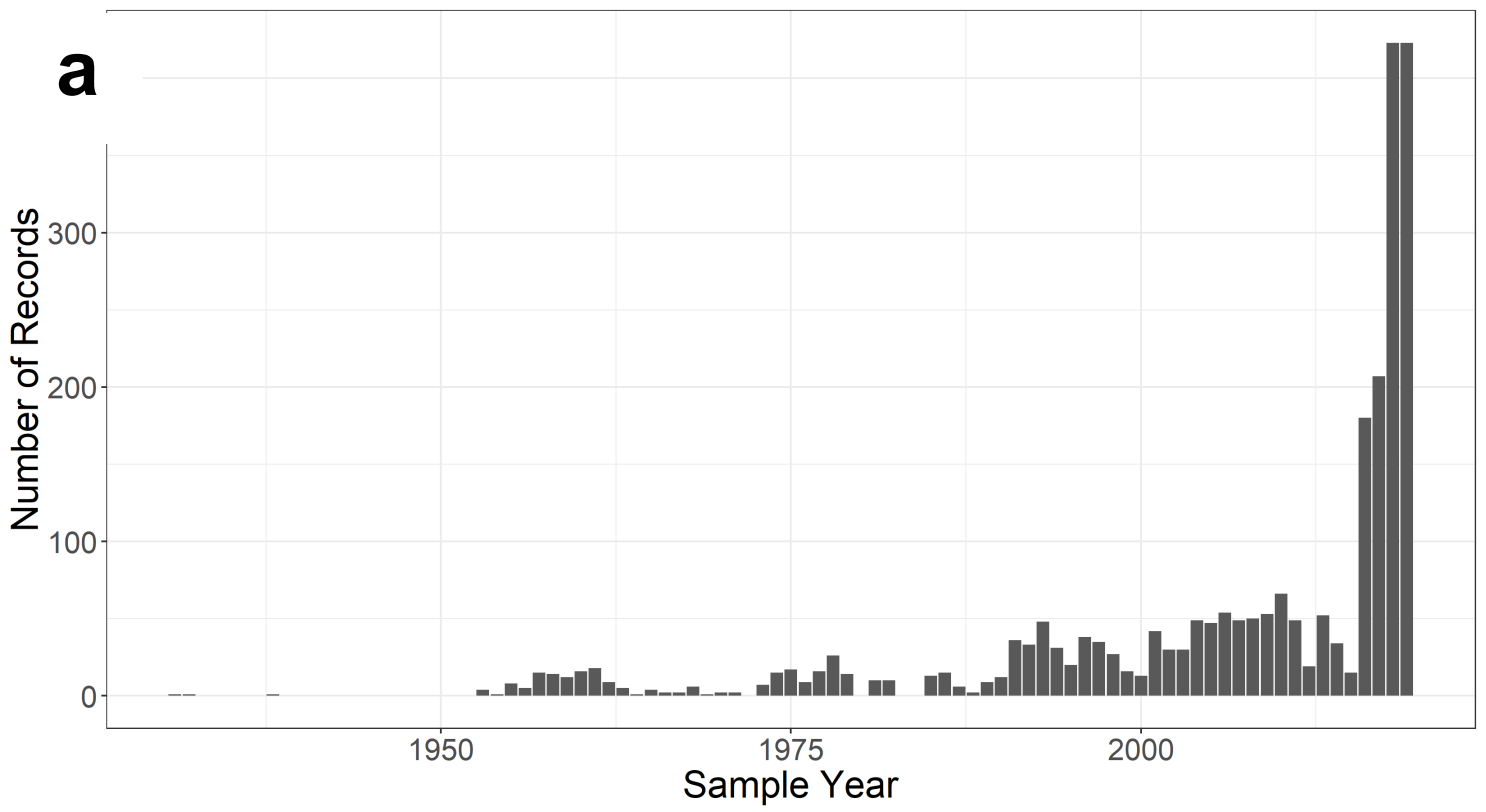
The number of species occurrence records for each year in the sampling period.

**Figure 7b.**
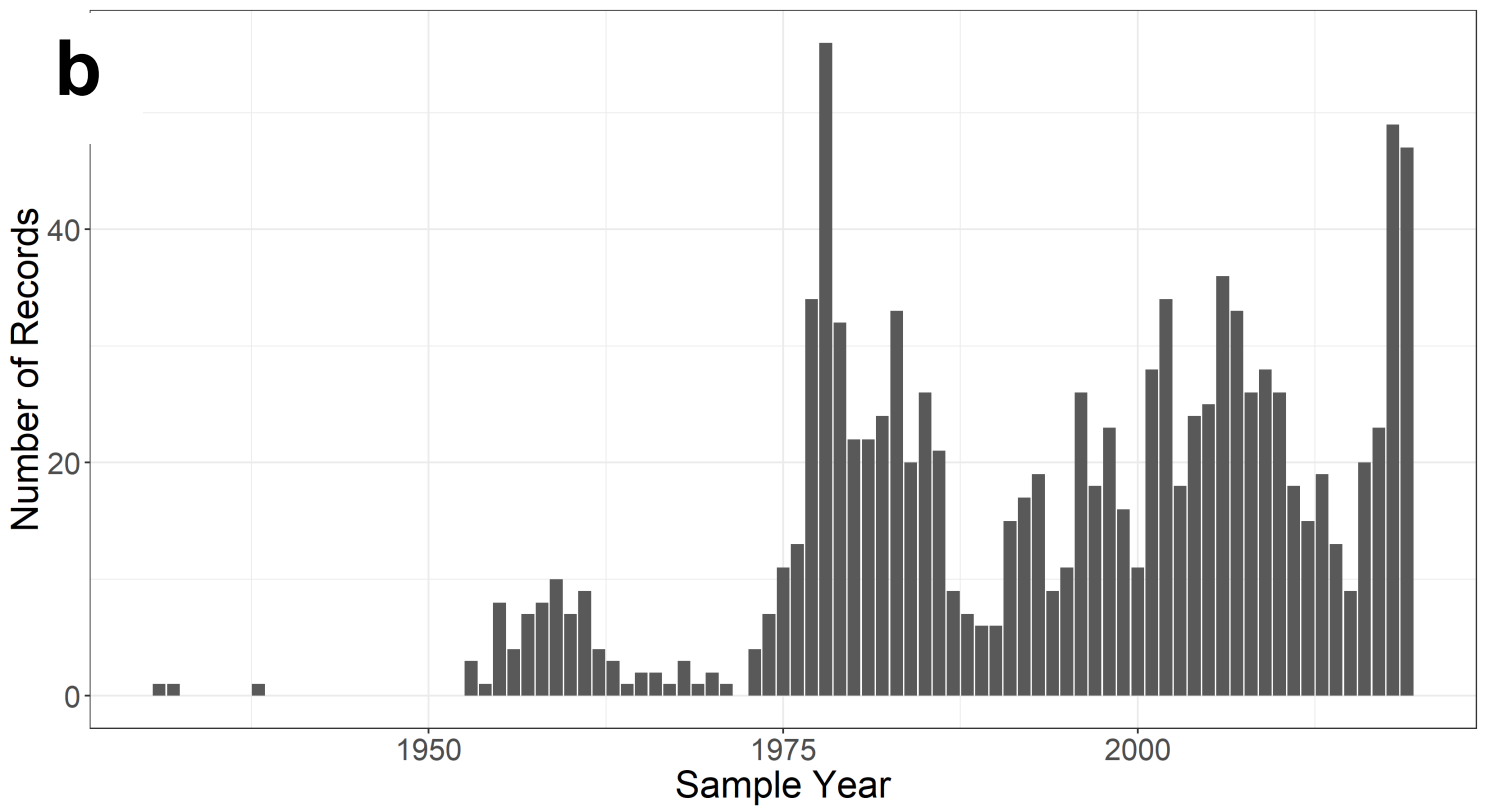
The number of environmental condition records for each year in the sampling period.
